# Pediatric vs. Adult Invasive Aspergillosis in Cancer and Hematopoietic Transplant Patients: Insights from a Matched Cohort at a Tertiary Cancer Center

**DOI:** 10.3390/jof11110771

**Published:** 2025-10-27

**Authors:** Saliba Wehbe, Ramia Zakhour, Ray Hachem, Ying Jiang, Hiba Dagher, Roseen Salman, Anne-Marie Chaftari, Issam I. Raad

**Affiliations:** 1 Department of Infectious Diseases, Infection Control and Employee Health, The University of Texas MD Anderson Cancer Center, Houston, TX 77030, USA; rhachem@mdanderson.org (R.H.); yijiang@mdanderson.org (Y.J.); achaftari@mdanderson.org (A.-M.C.); iraad@mdanderson.org (I.I.R.); 2 Division of Infectious Diseases, Department of Pediatrics, McGovern Medical School at UTHealth, Houston, TX 77030, USA; ramia.g.zakhour@uth.tmc.edu; 3 Department of Pediatrics, Division of Hematology-Oncology, Baylor College of Medicine, Houston, TX 77030, USA; rnsalman@bcm.edu

**Keywords:** invasive aspergillosis, pediatric invasive aspergillosis

## Abstract

**Background:** Invasive aspergillosis (IA) is a life-threatening infection in immunocompromised patients, including those with hematologic malignancies and hematopoietic stem cell transplants. While adult IA has been well characterized, data on pediatric populations remain limited, and potential age-related differences are often overlooked in current management guidelines. **Methods:** We conducted a retrospective matched cohort study at a tertiary cancer center, evaluating IA cases diagnosed over a 31-year period. Pediatric patients (≤18 years) with proven or probable IA were matched 1:3 with adult IA cases based on year of diagnosis, underlying disease, and history of hematopoietic cell transplantation. We compared demographics, clinical presentation, diagnostic modalities, microbiology, antifungal prophylaxis, and treatment approaches between the two groups. **Results:** The study included 34 pediatric and 102 matched adult IA cases. Pediatric patients were significantly more likely to present with neutropenia (*p* = 0.04) and sinus involvement (*p* = 0.048). Serum galactomannan testing was more often positive in pediatric patients (*p* = 0.027), whereas bronchoalveolar lavage galactomannan was more frequently positive in adults (*p* = 0.003). Differences in antifungal prophylactic regimens were also observed. **Conclusions:** Our findings underscore significant age-related variations in IA epidemiology, diagnostics, and management. These results support the development of age-specific diagnostic algorithms and antifungal strategies.

## 1. Introduction

Invasive aspergillosis (IA) continues to pose a significant threat to immunocompromised pediatric patients, particularly those undergoing chemotherapy, hematopoietic stem cell transplantation (HSCT), or receiving immunosuppressive therapy [1]. Despite advancements in antifungal therapies and diagnostic modalities, IA continues to be associated with high morbidity and mortality across all age groups [1,2,3]. While the underlying risk factors for IA, such as prolonged neutropenia, corticosteroid use, and immunosuppressive therapies, are common to both pediatric and adult populations, emerging evidence suggests notable age-related differences in the epidemiology, clinical presentation, diagnostic challenges, and management strategies of IA [1,4]. These disparities are clinically significant, yet pediatric-specific data remain limited, often necessitating the extrapolation of adult-based guidelines to younger patients [4,5].

One of the primary challenges in managing pediatric IA lies in its diagnosis. Non-culture-based diagnostic assays, including serum galactomannan (GM) and β-D-glucan (BDG), which are integral to IA diagnosis, vary in performance metrics between age groups [6,7].

Therapeutic approaches to IA also differ between children and adults, influenced by pharmacokinetic and pharmacodynamic variations [8]. For instance, voriconazole, a first-line antifungal agent for IA, exhibits faster clearance rates in children, necessitating higher or more frequent dosing to achieve therapeutic levels [8,9]. Limited availability of suitable oral formulations for younger children and delayed regulatory approval of new medications for pediatric patients contribute to additional differences between adult and pediatric treatment and prevention strategies, and limit antifungal options for children.

Antifungal prophylaxis practices further highlight age-related differences. Children undergoing cancer treatment or HSCT are less likely to receive mold-active azole prophylaxis compared to adults, potentially influencing IA incidence and outcomes [4,10]. These prophylactic disparities may also affect the epidemiology of *Aspergillus* species encountered, with some studies noting a higher prevalence of non-fumigatus aspergillosis including *Aspergillus flavus* in pediatric cases [11,12].

Outcomes of IA in pediatric patients remain a subject of ongoing investigation. While some studies suggest comparable or even better survival rates in children than adults, others report high mortality, particularly in specific subgroups such as HSCT recipients [13,14]. This data highlights the necessity for comprehensive, age-stratified analyses to better inform clinical decision-making.

Given the evolving understanding of age-related differences in invasive aspergillosis, this study aimed to compare the epidemiology, diagnostic approaches, treatment strategies, and clinical outcomes of IA in pediatric versus adult patients undergoing cancer therapy or HSCT. Using a matched cohort from a tertiary cancer center, we sought to identify age-specific patterns that could inform more personalized management guidelines and ultimately enhance care across the lifespan.

## 2. Materials and Methods

### 2.1. Study Design and Patient Selection

We conducted a retrospective matched cohort study of patients diagnosed with invasive aspergillosis at The University of Texas MD Anderson Cancer Center between July 1993 and April 2024. IA was classified as proven or probable according to the European Organization for Research and Treatment of Cancer and the Mycoses Study Group Education and Research Consortium (EORTC/MSGERC) criteria [15].

Within this period, we identified 34 pediatric patients (aged ≤ 18 years at diagnosis) with IA. Each pediatric case was matched with three adult patients (aged ≥ 19 years) from a pre-existing IA database, resulting in a 1:3 pediatric-to-adult ratio. Matching was based on the year of IA diagnosis (±1 year), cancer type (hematologic malignancy vs. solid tumor), and history of hematopoietic cell transplantation within one year prior to IA diagnosis. Adult comparators were selected through simple random sampling to ensure representativeness and minimize selection bias.

### 2.2. Data Collection

Clinical and demographic data were extracted from electronic medical records. Variables included patient age, sex, underlying malignancy, neutropenia status at the time of IA diagnosis, site of infection, galactomannan (GM) testing and culture results, treatment regimens, and outcomes.

Neutropenia was defined as an absolute neutrophil count (ANC) ≤ 500 cells/μL, and neutrophil recovery was defined as sustained ANC > 500 cells/μL for at least seven consecutive days. Sites of infection (e.g., pulmonary, sinus, central nervous system) were recorded based on clinical, microbiological, and imaging data documented at the time of diagnosis.

The primary outcomes were all-cause mortality and IA-attributable mortality at 6 and 12 weeks following diagnosis. IA-attributable mortality was defined as death occurring without evidence of clinical improvement and with no alternative cause identified. Therapeutic response at the end of antifungal therapy was also assessed, based on predefined institutional criteria that integrated clinical and microbiological evidence of resolution.

### 2.3. Data Management and Ethical Considerations

Data were securely entered into REDCap version 15.3.1, a web-based research data management platform. The study protocol was approved by the Institutional Review Board (IRB) at MD Anderson Cancer Center, PA17-0883 2023-10-17. Given the retrospective nature of the study and the use of de-identified data, informed consent was waived by the IRB.

### 2.4. Statistical Analysis

Chi-square or Fisher’s exact tests were used to compare categorical variables, as appropriate. The Wilcoxon rank-sum test was used to compare continuous variables. All tests were two-sided, with a significance level set at 0.05. Data analyses were performed using SAS version 9.4 (SAS Institute Inc., Cary, NC, USA).

## 3. Results

### 3.1. Demographics and Risk Factors

We identified 34 pediatric patients (≤18 years) and matched them with 102 adult patients diagnosed with proven or probable invasive aspergillosis between 1993 and 2024. Pediatric patients had a median age of 14 years (range: 2–18), while adult patients had a median age of 54 years (range: 19–84). The gender distribution was not significantly different, although a higher proportion of children were male (71% vs. 54% in adults, *p* = 0.088).

Most patients (91%) had a hematologic malignancy, and approximately 40% of pediatric patients had undergone hematopoietic stem cell transplantation. Neutropenia at diagnosis was significantly more common in pediatric patients compared to adults (64% vs. 43%, *p* = 0.04), likely reflecting differences in treatment regimens or baseline immune suppression. Patterns of antifungal prophylaxis also differed notably: pediatric patients were more likely to be receiving prophylaxis at the time of diagnosis yet were less likely to be on mold-active azoles and more likely to be receiving echinocandins (34% vs. 10% in adults, *p* = 0.002) (Table 1).

### 3.2. Disease Characteristics and Diagnostics

Pulmonary involvement was the most common site of infection overall but was more frequently seen among adults (92% vs. 70% in children, *p* = 0.003). In contrast, sinus involvement was more frequent in pediatric patients (9% vs. 1%, *p* = 0.048). A total of three pediatric patients had sinus infections, one of which was caused by *A. flavus*.

Aspergillus galactomannan (GM) testing was performed in a subset of patients. Serum GM was conducted in 24% of adult patients and 47% of pediatric patients, while BAL GM testing was conducted in 35% of adults and 32% of pediatric patients. Serum galactomannan was more commonly positive among pediatric patients (88% vs. 54%, *p* = 0.027), whereas BAL galactomannan positivity was more frequently observed in adults (61% vs. 9%, *p* = 0.003), potentially reflecting differential diagnostic approaches or variations in disease localization.

Among culture-confirmed cases, *A. flavus* was the most isolated specie in pediatric patients (41%), followed by *A. fumigatus* (29%). In contrast, *A. fumigatus* predominated in adults (32%) (Table 2).

### 3.3. Treatment and Outcomes

Pediatric patients were more often treated with antifungal monotherapy (65% vs. 56% in adults), while combination therapy was more frequently used in adults.

Regarding outcomes, ICU admission and mechanical ventilation rates were similar between groups. At 12 weeks, all-cause mortality was higher among pediatric patients (47% vs. 37% in adults), as was IA-attributable mortality (38% vs. 25%). However, these differences did not reach statistical significance.

## 4. Discussion

This matched cohort study provides a comprehensive comparison of invasive aspergillosis in pediatric and adult patients with cancer or hematopoietic stem cell transplantation over a 31-year period at a tertiary cancer center. The findings underscore notable age-related differences in clinical presentation, diagnostic approaches, antifungal prophylaxis, and outcomes, all of which point to the need for age-specific strategies in the management of IA.

Pediatric patients more frequently presented with neutropenia at the time of IA diagnosis, a finding that may reflect their higher exposure to intensive chemotherapy regimens or conditioning protocols for HSCT. Moreover, sinus involvement was significantly more common among pediatric patients (9% vs. 1%), a difference that may be attributed to anatomical factors, immune response variability, or environmental exposures that differ with age. In contrast, pulmonary IA was predominant in adults, consistent with literature emphasizing the lungs as the most common site of IA in immunocompromised adults [16]. In our cohort, pulmonary involvement was observed in 68% of pediatric cases, while another study reported pulmonary involvement in 40% of cases [17].

Differences in diagnostic outcomes were also observed between the two groups. Serum galactomannan testing was more frequently positive in children (88% vs. 54%), while BAL GM positivity was significantly higher in adults (61% vs. 9%). These differences may be influenced by the relative distribution of disease (e.g., more extrapulmonary involvement in children), the feasibility of conducting invasive procedures such as bronchoscopy in pediatric populations, and differing thresholds for initiating diagnostic testing. Prior studies have highlighted the limited sensitivity and specificity of galactomannan testing in children, which may stem from age-related differences in fungal burden, immune response, and diagnostic timing [6,18].

Antifungal prophylaxis strategies also differed notably between the groups. At the time of IA diagnosis, children were less likely receiving mold-active azole prophylaxis and more likely to be on echinocandins (34% vs. 10%). This may reflect ongoing concerns regarding azole pharmacokinetics in pediatric populations, including faster drug clearance, the need for higher dosing, and challenges related to therapeutic drug monitoring [9,19]. Echinocandins, though not first-line therapy for IA treatment, are often favored in pediatric patients for their safety profile and more predictable pharmacokinetics [20].

Microbiological findings revealed that *A. flavus* was isolated more frequently in pediatric patients (41%) than in adults (22%), while *A. fumigatus* remained the predominant species among adults. The higher prevalence of *A. flavus* in children has been documented in prior studies and may reflect differences in geographic exposure, host susceptibility, or local environmental conditions [11,12]. Other pediatric studies have reported *A. fumigatus* as the most commonly isolated species in children [21,22].

In our cohort, differences in mortality between pediatric and adult patients did not reach statistical significance. This finding is noteworthy, especially in light of previous studies that have reported improved survival outcomes in pediatric populations [23,24]. The increased mortality observed among pediatric patients in our study may be attributed to delayed recognition of IA, less frequent use of mold-active azole prophylaxis, differences in pharmacokinetics affecting antifungal efficacy, or more aggressive underlying malignancies.

This study’s retrospective design and single-center setting introduce inherent limitations, including possible changes in diagnostic criteria, and treatment protocols over time. Furthermore, the relatively small pediatric sample size may limit the statistical power to detect significant differences in outcomes or risk factors.

## 5. Conclusions

This matched cohort study underscores important age-related differences in invasive aspergillosis among cancer and HSCT patients. Pediatric patients were more likely to present with neutropenia, and sinus involvement. Diagnostic patterns also differed, with higher serum galactomannan positivity in children and more BAL galactomannan positivity in adults. These findings highlight the need for tailored diagnostic and therapeutic strategies in pediatric IA and support further prospective, age-stratified studies to inform evidence-based clinical guidelines.

## Figures and Tables

**Table 1:** Comparison of baseline characteristics of adult and pediatric patients with invasive aspergillosis.

Variables	Adult	Pediatric	*p*-Value
	(n = 102)	(n = 34)	
	N (%)	N (%)	
Age (years), median (range)	54 (19–84)	14 (2–18)	
Time period of IA diagnosis			0.926
1993–2003	18 (18)	6 (18)	
2004–2014	18 (18)	7 (20 *)	
2015–2024	66 (64 *)	21 (62)	
Gender			0.088
Male	55 (54)	24 (71)	
Female	47 (46)	10 (29)	
Type of cancer			>0.99
Hematologic malignancy	93 (91)	31 (91)	
Solid tumor	9 (9)	3 (9)	
Type of hematologic malignancy			
Leukemia	63/93 (68)	29/31 (94)	**0.005**
Lymphoma	17/93 (18)	2/31 (6)	0.153
Myeloma	6/93 (6)	0/31 (0)	0.335
Other	7/93 (8)	0/31 (0)	0.191
HSCT	36 (35)	14 (41)	0.538
Type of HSCT			0.418
Autologous	7/35 (20)	1/13 (8)	
Allogeneic	28/35 (80)	12/13 (92)	
GVHD	18 (18)	8 (24)	0.450
Neutropenia at diagnosis	43/100 (43)	21/33 (64)	**0.040**
Neutropenia recovery	13/41 (32)	12/21 (57)	0.053
Prophylaxis at diagnosis ^$^	58 (57)	26 (76)	**0.042**
Fluconazole	9/96 ^#^ (9)	5/32 ^#^ (16)	0.337
Voriconazole	9/96 ^#^ (9)	4/32 ^#^ (13)	0.736
Posaconazole	16/96 ^#^ (17)	4/32 ^#^ (13)	0.574
Isavuconazole	2/96 ^#^ (2)	0/32 ^#^ (0)	>0.99
Echinocandin	10/96 ^#^ (10)	11/32 ^#^ (34)	**0.002**
Aspergillosis classification			0.076
Proven	16/96 (17)	10/32 (31)	
Probable	80/96 (83)	22/32 (69)	
Type of IA infection ^^^			
Invasive pulmonary infection	91/99 (92)	23/33 (70)	**0.003**
Disseminated infection	2/99 (2)	3/33 (9)	0.099
Localized infection	6/99 (6)	5/33 (15)	0.142
Sinus infection	1/99 (1)	3/33 (9)	**0.048**

Abbreviations: IA: Invasive Aspergillosis, HSCT: Hematopoietic Stem Cell Transplant, GVHD: Graft Versus Host Disease. Note: * The percentage was slightly adjusted to ensure the group total sums to 100%. ^#^ Reflects the number of patients included in the analysis after excluding those with missing data regarding the specific type of prophylaxis received. ^$^ A few patients received more than one type of prophylaxis, and not all types of prophylactic therapy patients received are listed here. ^^^ There patients were excluded from the analysis due to missing data on the type of infection, and two patients in the analysis (one in each group) had more than one type of IA infection.

**Table 2:** Comparison of diagnostic findings, treatment, and clinical outcomes in adult and pediatric patients with invasive aspergillosis.

Variables	Adult	Pediatric	*p*-Value
	(n = 102)	(n = 34)	
	N (%)	N (%)	
Galactomannan diagnosis			
Positive serum GM *	13/24 (54)	14/16 (88)	**0.027**
Positive BAL GM ^#^	22/36 (61)	1/11 (9)	**0.003**
Positive serum and BAL GM	2/20 (10)	0/9 (0)	>0.99
Positive fungal culture	64 (63)	18 (53)	0.312
Aspergillus species ^$^			
* A. Fumigatus*	19/60 (32)	5/17 (29)	0.859
* A. Flavus*	13/60 (22)	7/17 (41)	0.125
* A. Terreus*	14/60 (23)	1/17 (6)	0.168
* A. Niger*	10/60 (17)	1/17 (6)	0.439
Other	6/60 (10)	3/17 (18)	0.405
Primary antifungal therapy			0.409
Monotherapy	51/91 (56)	20/31 (65)	
Combination therapy	40/91 (44)	11/31 (35)	
Type of primary monotherapy			
Amphotericin B	13/51 (25)	10/20 (50)	0.055
Azole	30/51 (59)	8/20 (40)	0.153
Echinocandin	8/51 (16)	2/20 (10)	0.714
ICU	41 (40)	17 (50)	0.317
Mechanical ventilation	31 (30)	10 (29)	0.914
Response at EOT	47/96 (49)	12/33 (36)	0.210
All-cause mortality			
At 6 weeks	33 (32)	13 (38)	0.530
At 12 weeks	38 (37)	16 (47)	0.312
Aspergillus-related mortality			
At 6 weeks	21/98 (21)	11/34 (32)	0.200
At 12 weeks	24/96 (25)	13/34 (38)	0.142

Abbreviations: GM: Galactomannan, BAL: Bronchoalveolar Lavage, ICU: Intensive Care Unit, EOT: End of Therapy. Note: * Positive result from serum GM test means either at least a single GM test with a value ≥ 1.0 or at least two tests with GM value ≥ 0.5 from both tests. ^#^ Positive result from BAL GM test means at least a single GM test with a value ≥ 1.0. ^$^ One adult patient was infected with three different *Aspergillus* species.

## Data Availability

The original contributions presented in this study are included in the article. Further inquiries can be directed to the corresponding author.
